# Correlation between spatial resolution and ball distortion rate of panoramic radiography

**DOI:** 10.1186/s12880-020-00472-5

**Published:** 2020-06-19

**Authors:** Han-Gyeol Yeom, Jo-Eun Kim, Kyung-Hoe Huh, Won-Jin Yi, Min-Suk Heo, Sam-Sun Lee, Soon-Chul Choi

**Affiliations:** 1 Department of Oral and Maxillofacial Radiology and Dental Research Institute, School of Dentistry, Seoul National University, 101 Daehak-ro, Jongno-gu, Seoul, 03080 South Korea; 2 Department of Oral and Maxillofacial Radiology, College of Dentistry, Daejeon Dental Hospital, Wonkwang University, Daejeon, South Korea

**Keywords:** Phantom, Radiology, Panoramic radiography, Ball distortion rate, Spatial resolution

## Abstract

**Background:**

The purpose of this study was to analyze the correlation between spatial resolution and ball distortion rate of panoramic radiography and to elucidate the minimum criterion for ball distortion rate, which is very relevant to clinical readability.

**Methods:**

Horizontal and vertical spatial resolution and ball distortion rates were calculated in the same position, such as the incisor, premolar, molar, and temporomandibular joint area with various object depths corresponding to 48 mm. Three devices were evaluated. A region showing spatial resolution above the reference standard was selected, and the ball distortion rate corresponding to the same region was divided into horizontal and vertical phantom groups.

The mean and standard deviation of the obtained ball distortion rates were calculated. Student’s t-test was used to statistically analyze the mean difference in ball distortion rates between vertical and horizontal phantom groups.

**Results:**

In all devices, the horizontal line pair phantom, but not the vertical line pair phantom, was readable in all areas measured at the line pair value of at least 1.88 lp/mm. The line pair value tended to be higher toward the center and lower toward the outside. The ball distortion rate tended to decrease closer to the center and increased further away. In addition, ball distortion rates could not be measured at some areas as they were not recognized as balls due to the high degree of distortion at the outermost and innermost sides. The number of balls satisfying the reference value using the horizontal line pair phantom was 102 (mean of ball distortion rates, 20.98; standard deviation, 15.25). The number of balls satisfying the reference value using the vertical line pair phantom was 49 (mean of ball distortion rates, 16.33; standard deviation, 14.25). However, mean ball distortion rate was not significantly different between the two groups.

**Conclusions:**

Image layer of panoramic radiography could be evaluated by the spatial resolution using horizontal and vertical line pair phantoms and by assessing ball distortion rates through a ball-type panorama phantom. A ball distortion rate of 20% could be used as a threshold to evaluate the image layer of panoramic radiography.

## Background

In dentistry, evaluation and diagnosis of hard tissue, such as the alveolar bone and teeth, are essential [1]. Therefore, radiation exposure of patients in dental clinics has been estimated to represent more than 25% of the total annual frequency of all medical radiographic examinations [2]. Panoramic radiography is a frequently used imaging modality in dental clinics [2, 3]. In addition to obtaining information about the entire hard tissue of the oral and maxillofacial regions from a single image, the image acquisition process is also simple and painless for patients [1, 3, 4].

Panoramic radiography is a technique combining the principles of scanning and tomography [5]. In panoramic radiography, a horizontally narrow slit X-ray beam is used to scan the oral and maxillofacial regions, and the obtained radiograph is recorded on a rotating single large film [4, 6]. Due to this acquisition process, the images of the structures located in the arch shaped plane among the three-dimensional areas are most clearly obtained [4, 6–9]. This vertical curved plane is called an image layer, sharply depicted layer, focal trough, or the machine’s zone of sharpness [4, 6–10]. Image sharpness gradually decreases as the objects move away from the center of the image layer, and images are no longer identifiable on the radiograph [11–13]. This unique characteristic is of clinical importance as the radiologists need to acquire diagnosing data for the oral and maxillofacial regions without any overlapping structures [12]. This is a crucial feature of panoramic radiography, which is related to its diagnostic potential and is the reason for its use in the dental field. However, at the same time, the complexity of the image acquisition process makes it difficult to evaluate the quality of a panoramic radiograph using conventional image quality assessment methods.

In several previous studies, the image layers of panoramic radiography have been evaluated; however, the evaluation methods and criteria varied. Object distortion rate or sharpness of the boundary on a panoramic image obtained using pins, screws, or balls has been studied [7, 8, 10, 13]. Theoretically, when the ball-shaped object of sufficiently small diameter is placed with its center point in the image layer, the ball-image will appear as undistorted round shape. Devlin et al. combined mathematical theory and practical approach using several sized metal balls [10]. They showed that the distortion is zero when the object is placed in certain positions and moving the object away from the points results in distortion [10]. Recently, a new ball-type phantom was proposed that can evaluate the shape and size of the image layer of panoramic radiography with single image acquisition [14]. Using the phantom in which the 704 balls with a 2-mm diameter are planted in a form that follows the shape of a dental arch at an object depth of 46 mm, images of 704 balls can be acquired at a single image acquisition run without object overlapping. However, it is necessary to set a threshold for a clinically diagnosable significant ball distortion rate.

The method of evaluating the image layer using lead line pairs was first presented by Hassen et al. [6]. Since then, there several studies have evaluated the image layer using the readability of the lead line pair [15, 16]. Moreover, in a previous study, the position of the arch (incisor, premolar, molar, and temporomandibular joint) was divided and the reference line pair value associated with the minimum image quality was presented for each position [17].

However, no study has matched the spatial resolution and distortion rate of the corresponding parts. In addition, there is no standard method for evaluating the image layer of panoramic radiography normalized to the thresholds of line pair value or distortion rate.

Therefore, the present study aimed to match the spatial resolution and ball distortion rates at the same position of panoramic radiography. In addition, through this correlation, the minimum criterion for the ball distortion rate, which will be the most relevant for clinical diagnosis, was suggested.

## Methods

### Determination of spatial resolution

Horizontal resolution phantoms of staircase shape were used to obtain images of horizontal lead line pairs in the region corresponding to a width of 48 mm at 4-mm intervals. They were located at the incisor, premolar, molar, and the temporomandibular joint (TMJ) areas. One phantom comprised 13 steps, each containing a pair of horizontal lead line pairs of the same value. Four horizontal resolution phantoms were used each for the lead line pairs of 1.88, 2.32, 2.58, and 3.19 lp/mm. These values were determined based on the IEC Standard 4, which uses lead line pairs of 1.6–3.0 lp/mm for evaluating panoramic radiography [18]. Nuclear Associates model 07–501 SER. NO.12913® (Fluke Co., Cleveland, OH, USA) was used, laser cut and placed at each step.

Four vertical resolution phantoms with vertical lead line pairs placed at an oblique angle to the same width of 48 mm were also designed, each for the lead line pairs of 1.88, 2.32, 2.58, and 3.19 lp/mm. Ligature wires were used at the boundary to mark the 4-mm interval horizontally.

To locate the prepared phantoms, an arch shaped phantom stand was used. It was designed to have square-shaped holes in four positions, incisor, premolar, molar, and TMJ. The data of the incisor, premolar (central points of the cusps of mandibular first premolar and mandibular second premolar), molar (central points of the mesiobuccal cusp of mandibular first molar and the distobuccal cusp of mandibular second molar), and TMJ were determined by previously developed ball type panorama phantom [14]. A space of 20 mm was left under the phantoms to overcome the limitation of overlapping images of the lead line pairs at the lowest step due to the phantom stand being placed on the opposite side of the actual image of the phantom itself to reduce the ghost image.

Panoramic radiographs of the phantoms were obtained using OP-100® (Instrumentarium Dental, Tuusula, Finland), PCH-2500® (Vatech, Gyeonggi, Korea), and Rayscan α-P® (Ray, Gyeonggi, Korea) at Seoul National University Dental Hospital, Seoul, South Korea. The images were obtained with optimal parameters according to the user manual for imaging adult males, which are regularly used in the department. The imaging parameters were 73 kVp, 10 mA, and 17.6 s for OP-100®; 73 kVp, 10 mA, and 13.5 s for PCH-2500®; and 73 kVp, 10 mA, and 14.0 s for Rayscan α-P®. The midpoint of the centerline of the phantom stand was arranged at the center of the incisive notch. A tripod water level was used to position these accurately. Attenuation by the skull was reproduced with an 0.8-mm copper plate on the X-ray source, according to the recommendations of IEC Standard 4 [18]. According to the standard, a 6-mm aluminum plate should be attached to the front of the phantom to mimic attenuation by soft tissue. However, the entire phantom could not be covered uniformly due to structural limitations such that it overlapped with the copper plate and was placed in the X-ray source. Images of the phantoms were obtained three times in the same position to compensate for errors in image acquisitions.

The images of resolution phantoms were evaluated twice by the consensus of two oral–maxillofacial radiologists with over 20 years of experience. Following the random arrangement of the images, the readable range numbers (1 ~ 13, numbered from the lowest area) were determined. Images were rearranged randomly after 2 weeks and reevaluated to minimize error. The images of one phantom resolution image were acquired three times, and each image was read twice, resulting in six results for each resolution phantom for one region. The value that could be read more than four times was determined as the readable line pair value for that area.

### Determination of ball distortion rate

The ball-type panorama phantom proposed in a previous study was used [14]. This phantom was designed to obtain 704 metal ball images without overlapping by acquiring a single image. The diameter of the balls was 2 mm. The balls were closely positioned in a 46-mm wide jawbone shape.

The same panoramic radiography systems (OP-100®, PCH-2500®, and Rayscan α-P®) and parameters were used. The midpoint of the centerline of the ball-type phantom was positioned at the center of the incisive notch. A tripod water level was also used. For the ball-type panorama phantom, additional bone and soft tissue attenuation was not reproduced to compensate for the X-ray attenuation caused by acrylic resin placed on the ball. The exposure was repeated three times to minimize errors.

A program was created using MATLAB® (Mathworks Inc., Natick, MM, USA) to analyze the obtained ball-type panoramic phantom images. To clearly emphasize the boundaries of the obtained images of the balls, a “noise reduction filter” was applied and then “ellipse detection” was applied with a “universal threshold” to detect the boundaries of the balls obtained in circular or elliptical shapes on the images. The lengths of the long and short axes of the balls were measured using the boundaries. The differences in ball distortion ratio of the two lengths was used as an index representing the degree of ball deformation (ball distortion rate).

$$ \mathrm{Ball}\ \mathrm{distortion}\ \mathrm{rate}\ \left(\%\right)=\left(|1-\frac{\mathrm{horizontal}\ \mathrm{length}\ \mathrm{of}\ \mathrm{the}\ \mathrm{obtained}\ \mathrm{ball}\ \mathrm{image}}{\mathrm{vertical}\ \mathrm{length}\ \mathrm{of}\ \mathrm{the}\ \mathrm{obtained}\ \mathrm{ball}\ \mathrm{image}}|\right)\times 100 $$

By applying various distortion rate thresholds from 5 to 50% at 5% increments, the lowest satisfied ball distortion rates were determined as the final-ball distortion rate. After the pixel size and distortion rate threshold were inserted, the MATLAB® program operated fully automatically.

### Data analysis

The theory behind the rationale of the study was that the resolution value at the same site evaluated by each of the horizontal and vertical line pair phantoms would show different results. However, the ball distortion rate of the part that satisfies clinically meaningful criteria (evaluated by each spatial resolution measurement method) will not show a significant difference in its mean value. Once this theory is validated, the reference ball distortion factor, which can satisfy both the vertical and horizontal diagnostic values, can be presented as a criterion for the image layer.

To analyze the ball distortion rate corresponding to the area showing clinically meaningful quality, the reference line pair values with clinically desirable qualities from the previous study were used [17]. The reference line pair values were 3.19 lp/mm in the incisor, 2.32 lp/mm in the premolar and TMJ, and 1.88 lp/mm in the molar region.

The ball distortion rates for the same site that satisfied the reference line pair values using a horizontal or vertical resolution phantom were selected and divided into the horizontal phantom and vertical phantom groups.

Statistical analysis was performed using SPSS 21.0® (IBM Corp., Armonk, NY, USA). Values for all parameters were expressed as numbers. Levene’s test was used to determine whether data were normally distributed, and Student’s paired t-test was used to compare the ball distortion rates satisfying each line pair value. A *P*-value of < 0.05 was considered significant.

## Results

**Fig. 1:**
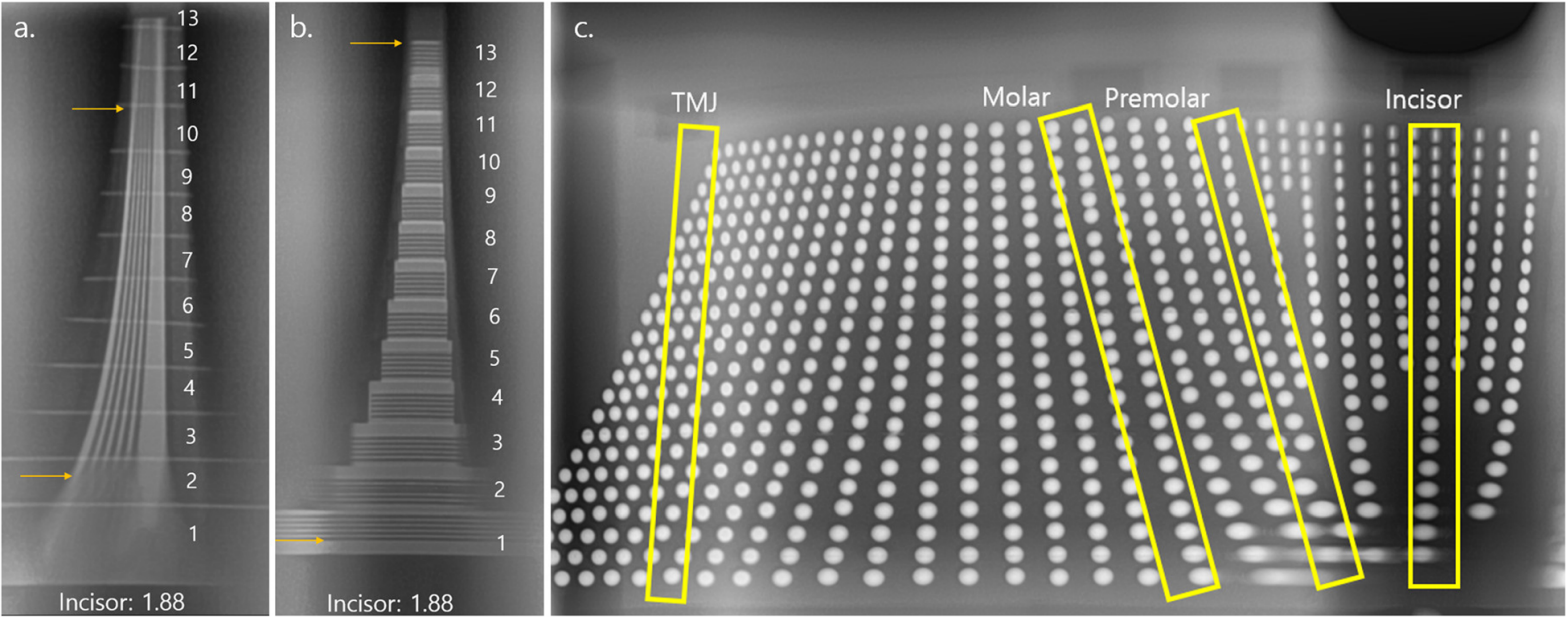
Panoramic radiographs of the phantoms using PCH-2500®. The readable area was evaluated twice by the consensus of two oral–maxillofacial radiologists with over 20 years of experience. (**a, b**). **a** Horizontal resolution phantom containing 1.88 lp/mm was located in the incisor region. The readable boundary was indicated by yellow arrows. (Step number: 1 ~ 13, numbered from the lowest area). **b** Vertical resolution phantom containing 1.88 lp/mm was located in the incisor region. The readable boundary was indicated by yellow arrows. (Step number: 1 ~ 13, numbered from the lowest area). c Panoramic radiograph of ball phantom. The incisor, premolar, molar, and TMJ regions used for analysis were indicated by yellow boxes

Panoramic radiographs used to measure the resolution of incisor region using PCH-2500® were shown in Fig. 1a-b. The measured values for the three systems are listed in Tables 1, 2, and 3, respectively. In all devices, the horizontal line pair phantom was readable in all areas at a line pair value of at least 1.88 lp/mm. In the incisor region, 3.19 lp/mm were readable in some parts in all 3 devices. However, the vertical line pair phantom was not readable at all line pair values in the outermost and innermost portions. The line pair value tended to be higher toward the center and lower toward the outside or inside. In many parts, the line pair value was lower when evaluated using the vertical line pair phantom than when evaluated using the horizontal line pair phantom.

**Table 1:** Test results for OP-100®

Position (Step)	Incisor			Premolar			Molar			TMJ		
	PP (%)	HR (lp/mm)	VR (lp/mm)	PP (%)	HR (lp/mm)	VR (lp/mm)	PP (%)	HR (lp/mm)	VR (lp/mm)	PP (%)	HR (lp/mm)	VR (lp/mm)
1	x	3.19	x	x	3.19	x	x	3.19	x	25	2.58	2.58
2	x	3.19	x	x	3.19	x	x	3.19	x	15	2.58	2.58
3	75	3.19	x	90	3.19	x	55	3.19	1.88	15	2.58	2.58
4	45	3.19	2.58	45	2.58	2.32	25	3.19	2.32	15	2.58	2.32
5	20	3.19	3.19	15	2.58	2.58	15	2.58	2.58	15	2.58	1.88
6	5	3.19	2.58	5	2.58	2.58	15	2.58	2.58	15	2.58	1.88
7	20	2.58	x	15	2.58	1.88	5	2.58	2.58	15	2.58	1.88
8	30	2.58	x	15	2.58	x	15	2.58	2.32	15	2.58	1.88
9	35	2.58	x	25	2.58	x	20	2.58	x	15	2.58	x
10	45	2.58	x	25	2.58	x	20	2.58	x	20	2.58	x
11	55	2.58	x	30	2.58	x	20	2.58	x	20	2.58	x
12	x	2.58	x	30	2.58	x	25	2.58	x	20	2.58	x
13	x	2.58	x	30	2.58	x	x	2.58	x	25	2.58	x

*PP* Ball distortion rate evaluated using panorama phantom, *HR* Spatial resolution evaluated using horizontal resolution phantom, *TMJ* temporomandibular joint, *VR* Spatial resolution evaluated using vertical resolution phantom

x: no line pair value could be read

**Table 2:** Test results for PCH-2500®

Position (Step)	Incisor			Premolar			Molar			TMJ		
	PP (%)	HR (lp/mm)	VR (lp/mm)	PP (%)	HR (lp/mm)	VR (lp/mm)	PP (%)	HR (lp/mm)	VR (lp/mm)	PP (%)	HR (lp/mm)	VR (lp/mm)
1	x	2.58	x	x	2.58	1.88	x	2.58	3.19	5	3.19	2.58
2	x	2.58	2.32	x	2.58	3.19	20	2.58	3.19	5	3.19	2.58
3	60	3.19	3.19	x	3.19	3.19	20	2.58	3.19	10	2.58	2.58
4	55	3.19	3.19	x	3.19	3.19	10	2.58	3.19	10	2.58	2.32
5	40	2.58	2.58	15	3.19	2.58	5	2.58	2.58	15	2.58	2.32
6	35	2.58	2.58	5	3.19	2.58	5	2.58	2.58	20	2.58	1.88
7	5	2.58	2.58	5	2.58	2.58	5	2.58	2.58	25	2.58	1.88
8	10	2.58	2.58	15	2.58	2.58	5	2.58	2.32	25	2.58	1.88
9	25	2.58	2.58	20	2.58	2.32	5	2.58	2.32	30	2.58	x
10	45	2.58	1.88	20	2.58	1.88	5	2.58	2.32	30	2.58	x
11	45	2.58	x	35	2.58	1.88	5	2.58	2.32	35	2.58	x
12	50	2.58	x	35	2.58	1.88	5	2.58	2.32	40	2.58	x
13	55	2.58	x	35	2.58	1.88	x	2.58	2.32	45	2.58	x

*PP* Ball distortion rate evaluated using panorama phantom, *HR* Spatial resolution evaluated using horizontal resolution phantom, *TMJ* temporomandibular joint, *VR* Spatial resolution evaluated using vertical resolution phantom

x: no line pair value could be read

**Table 3:** Test results for Rayscan α-P®

Position (Step)	Incisor			Premolar			Molar			TMJ		
	PP (%)	HR (lp/mm)	VR (lp/mm)	PP (%)	HR (lp/mm)	VR (lp/mm)	PP (%)	HR (lp/mm)	VR (lp/mm)	PP (%)	HR (lp/mm)	VR (lp/mm)
1	x	3.19	x	x	2.32	x	x	2.58	x	20	3.19	2.32
2	x	3.19	x	x	2.32	x	45	2.58	x	10	2.58	2.32
3	x	3.19	x	x	2.58	x	40	2.58	1.88	5	2.58	2.32
4	x	3.19	2.58	45	2.58	2.32	30	2.58	3.19	5	2.58	2.58
5	x	2.58	3.19	15	2.58	2.58	10	2.58	3.19	10	2.58	x
6	x	2.58	2.58	10	2.58	2.58	5	2.58	2.58	10	2.58	x
7	40	2.58	1.88	5	2.58	1.88	10	2.58	1.88	10	2.58	x
8	5	2.58	1.88	20	2.58	x	15	2.58	x	15	2.58	x
9	15	2.58	x	20	2.58	x	20	2.58	x	x	2.58	x
10	20	2.58	x	20	2.58	x	20	2.58	x	x	2.58	x
11	45	2.58	x	25	2.58	x	20	2.58	x	x	2.58	x
12	x	2.58	x	30	2.58	x	20	2.58	x	x	2.58	x
13	x	2.58	x	30	2.58	x	x	2.58	x	x	2.58	x

*PP* Ball distortion rate evaluated using panorama phantom, *HR* Spatial resolution evaluated using horizontal resolution phantom, *TMJ* temporomandibular joint, *VR* Spatial resolution evaluated using vertical resolution phantom

x: no line pair value could be read

Panoramic radiograph of ball phantom using PCH-2500® was presented in Fig. 1c. The ball distortion rate tended to decrease as closer to the center and increased further away. Ball distortion rates in some areas were not measured as they were not recognized as balls due to the high degree of distortion on the outermost and innermost sides.

Overall, the line pair value tended to be lower and the ball distortion rate tended to be higher when moving from the central part to the outside or inside regions. The line pair value or ball distortion rate could not be measured for some of the outermost and innermost regions.

The number of balls that met phantom resolution criterion was 102 and 49 in the horizontal and vertical phantom groups, respectively. Mean (±SD) ball distortion rate was 20.98 (±15.25) in the horizontal phantom group, 16.32 (± 4.25) in the vertical phantom group, and 19.47 (±15.10) in both groups.

In Levene’s test, the *p*-value between the two groups was 0.753 (i.e., > 0.05); thus, equal variances were assumed. In Student’s t-test, the p-value was 0.076, and the mean rate was not significantly different between the two groups. The mean difference was 4.65, with the standard error of the mean of 2.61. The results of the statistical analyses are listed in Table 4.

**Table 4:** Results of Levene’s test and Student’s t-test between the horizontal phantom and vertical phantom groups

Levene’s test		Student’s t-test						
F	Sig.	t	df	p (two-tailed)	Mean difference	Std. error difference	95% confidence interval of the Difference	
							Lower	Upper
0.099	**0.753**	1.786	149	**0.076**	4.65386	2.60591	−0.49545	9.80318

Statistical significance at *p* < 0.05

## Discussion

With advances in diagnostic radiation modalities, various standards have been established for the optimization of equipment as problems with radiation exposure have emerged [18, 19]. However, in the case of panoramic radiography, which is the most commonly used imaging modality in dentistry, it is difficult to apply uniform standards due to the complexity of the equipment.

Panoramic radiography forms the image layer along with the shape of the jawbone and shows only the image of the object located in the image layer clearly [20]. This is the area in which images with the highest spatial resolution and the lowest distortion can be obtained among the three-dimensional areas that the X-rays pass through [21]. Accurate evaluation of this area and assessments of whether the shape matches the overall jaw shape of actual subjects and whether its resolution and distortion are appropriate are essential for the determining the efficacy of panoramic radiation equipment [20].

In this study, theoretically, the image layer was the region with the best spatial resolution and the least distortion rate. We aimed to assess whether the image layers, evaluated by the two most common concepts, actually paired. Moreover, in the evaluation of spatial resolution, the horizontal and vertical line pair phantoms were separately used and analyzed considering that the horizontal and vertical factors are differently affected due to the characteristics of the equipment.

The position of image layer is dependent on many factors. These factors include the angular movement of the x-ray beam during exposure, focus-film distance, width of the x-ray beam, focal spot size [4]. Therefore, in the analysis conducted on three devices, each showed different results; however, the overall horizontal and vertical line pair values were higher and the ball distortion rates were lower near the arch-shaped center line (step 7). These results are consistent with the premise that the closer the objects located to the image layer, the higher the spatial resolution and the smaller the distortion rate. Overall, the line pair value was lower when evaluated using the vertical line pair phantom than when evaluated using the horizontal line pair phantom. When using vertical line pair phantom, the value is determined using the visibility of the horizontal gap of vertically positioned lines. The horizontal factor measured by the vertical line pair phantom may be much more sensitive to the positional change than the vertical factor because of the rotating feature of the panoramic radiograph.

**Fig. 2:**
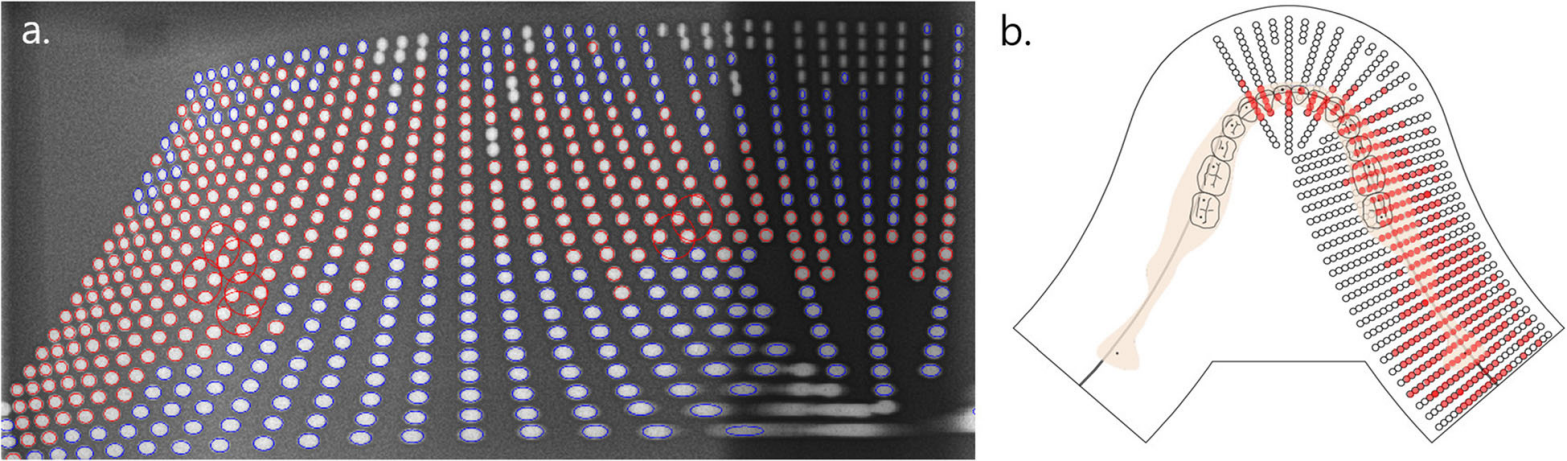
The suggested image layer by applying ball distortion rate thresholds (20%) in OP-100®. **a** The balls satisfying ball distortion rate threshold are shown in red, and the balls that did not satisfy that threshold are shown in blue. **b** The balls shown in red were matched to their original position. Since the average jaw shape is well located in the image layer, it is considered that this device is suitable for taking diagnostic images

Statistical analyses in this study used the criteria defined in previous studies presenting the minimum reference line pair values to obtain clinically desirable image qualities. The hypothesis was that the ball distortion rate evaluated by different resolution phantoms would not show a significant difference. The ball distortion rates for the same site that satisfy the reference line pair value when using the horizontal or vertical resolution phantom were extracted and the mean ball distortion rate that met any of the criteria was 19.47 ± 15.10. Student’s t-test indicated no significant difference in the mean ball distortion rate between the two groups (*p* = 0.076). This means that the line pair value at each area measured by the horizontal and vertical line pair phantoms was different; however, there was no significant difference in ball distortion rate at a site showing a greater clinically readable diagnostic quality. In previous studies that analyzed ball or object (e.g., pin or screw) distortion in panoramic radiation equipment, no suggestion of the range or threshold for the distortion level was set to obtain clinically readable high-quality images [7, 8, 14, 21]. In this study, using the spatial resolution reference, the mean of ball distortion rate of the readable area was 19.47. As the ball distortion rate was measured in 5% increments to minimize the error caused by the size of the ball and the pixel, the minimum ball distortion rate, 20%, which is the most relevant to clinical readability, could be suggested. The suggested image layer by applying ball distortion rate thresholds of 20% were presented in Fig. 2. The narrow incisor region of the image layer means that this region would be more easily distorted through malpositioning of the patient.

The standard presentation of the image layer through the ball distortion rate facilitates the evaluation of the image layer for various parts and also has the advantage that the image layer can be evaluated without subjective human evaluation of readability. However, requirement of the MATLAB® coding program for analysis may limit the application of this approach. Nonetheless, by developing the same method using the C# programing language, it would be easy to evaluate panoramic radiography results even at a local dental clinic with a Windows-based computer.

If the image layer of the panoramic radiograph can be evaluated simply and accurately, it can provide additional information to assist accurate diagnosis and can also help set a standard for obtaining high-quality images. Radiation exposure can be optimized through the establishment and application of appropriate evaluation methods and policies. By taking all of this into consideration, our approach will help minimize patients’ radiation exposure and maintain their health and safety.

## Conclusions

Image layer of panoramic radiography could be evaluated by the spatial resolution using horizontal and vertical line pair phantoms and by assessing ball distortion rates through a ball-type panorama phantom. When the image layer was inferred with the spatial resolution suggested as the minimum criterion for obtaining clinically readable images from previous studies, the corresponding mean ball distortion rate was measured to be 19.47. Therefore, a ball distortion rate of 20% could be proposed as a threshold to evaluate the image layer of panoramic radiography.

## Data Availability

All data generated or analyzed during this study are included in this published article.

## Abbreviations

TMJ: Temporomandibular joint
PP: Ball distortion rate evaluated using panorama phantom
HR: Spatial resolution evaluated using horizontal resolution phantom
VR: Spatial resolution evaluated using vertical resolution phantom
